# Relationship Amongst Technology Use, Work Overload, and Psychological Detachment from Work

**DOI:** 10.3390/ijerph16234602

**Published:** 2019-11-20

**Authors:** Juan Sandoval-Reyes, Julio C. Acosta-Prado, Carlos Sanchís-Pedregosa

**Affiliations:** 1 Academic Department of Social Psychology and Organizations, Faculty of Psychology, Universidad de la Sabana, 250001 Cundinamarca, Colombia; juan.sandoval1@unisabana.edu.co; 2 Faculty of Business Science, Universidad del Pacífico, Lima, Peru; jc.acostap@up.edu.pe or; 3 Faculty of Management, Universidad Externado de Colombia, Bogota, Colombia; 4 Faculty of Economic and Business Sciences, Universidad de Sevilla, 41004 Seville, Spain

**Keywords:** technology use, work overload, psychological detachment, psychological well-being, PLS-SEM

## Abstract

Permanent connection to the work world as a result of new technologies raises the possibility of workday extensions and excessive workloads. The present study addresses the relationship between technology and psychological detachment from work resulting from work overload. Participants were 313 professionals from the health sector who responded to three instruments used in similar studies. Through PLS-SEM, regression and dependence analyses were developed, and through the bootstrapping method, significance of factor loadings, path coefficients and variances were examined. Results of the study corroborate a negative effect of technology use on psychological detachment from work and a positive correlation between technology and work overload. Additionally, there is a significant indirect effect of technology on psychological detachment from work as a result of work overload. Findings extend the literature related to the stressor-detachment model, and support the idea that workers who are often connected to their jobs by technological tools are less likely to reach adequate psychological detachment levels. Implications for the academic community and practitioners are discussed.

## 1. Introduction

Socio-economic changes happen all over the world. Multiple new technologies and a progressive increase in competitiveness among companies are factors that inevitably end up influencing organizational contexts and hence challenging manager’s and worker’s skills [1]. The modern era demands faster, more-efficient, competitive organizations in order to survive in the globalized world [1,2], which in turn leads to a work intensification. As a result, new labor dynamics imply bigger demands on people, since it is increasingly expected that employees achieve greater and faster production.

Technology plays an important role in communication and collaboration at work. However, it is not the only way that technology participates in the business world. Although it skips the scope of the present study, manufacturing technologies have a strong impact on a company productivity. This view inevitably gives rise to a paradox that points out a two-fold impact of technology. While technological tools are included in an organization to hasten different processes, they may also demand increased effort from employees. As a consequence, there are new requirements in regards to handling these technologies, adjusting to them, maintaining upgraded versions, and balancing them with employees’ workloads, the latter requirement being the focus of this study.

These new demands require that people increase their efforts to achieve the demanding performance objectives that correspond to the business models of a globalized world [3]. Therefore, people should increase their emotional effort, as well as levels of mental and psychological activation, which can lead to the depletion of resources and affect the process of recovery from work demands [4,5]. It is required that the physical and psychological state of the person remain optimal, ensuring adequate levels of energy, motivation and commitment [6,7].

In this scenario, technological tools are increasingly acquiring more functionalities so that workers can respond to current demands. Many organizations aiming to improve productivity are developing time and space flexibility strategies for labor activities with the aid of technological support. In this sense, the possibility of a permanent connection to work thanks to developments in information and communication technologies implicitly implies an extension of the work day [8]. This can lead workers to an unwanted state that can be called “always in work mode” [9] and that assumes ever-increasing workloads.

Some studies find that work overload is a characteristic of job satisfaction, despite leading to unhealthy patterns [8] mediated in turn by a desire for incentives, security status and the ability to be promoted [10,11]. Nonetheless, extensive literature shows that work overload is negatively related to health indicators [12,13]. Hence, work overload manifests itself as a stressor that may have an impact on the levels of recovery from long working hours and, as a result, low psychological detachment.

Our study explores the possible relationship between the technology use and psychological detachment from work mediated by work overload. In this sense, we present our main constructs in the following sections: technological use, work overload, and psychological detachment.

### 1.1. Technology Use

New technologies have generated great benefits for companies by facilitating and encouraging employees to carry on work activities outside the physical environment of a traditional office. Technological tools continuously achieve higher levels of agility, dynamism and accessibility, allowing people to remain constantly connected to their work [14]. People are in a continuous state of attention to labor issues and reflecting on past or present problems, or on future opportunities related to their job performance [15].

A person’s possibility to be connected to their work environment all the time is positive, since they can have information in real time, be aware of possible contingencies and respond more agilely to problems that may arise [16]. Despite its benefits, the intensive use of information and communication technologies as management tools, dilutes the boundaries between physical spaces and work responsibilities. Furthermore, this can lead to extended working hours, making it more difficult for people to psychologically detach themselves from their work during break times. Likewise, the use of technologies prolongs exposure to work demands beyond the workday, wasting resources for recovery and exposing workers to labor demands for a longer period of time [17].

### 1.2. Work Overload

Paskvan and Kubicek [1] indicate that work overload is characterized by the need to work faster, the need for quicker responses, reduced break periods and the need to perform multiple tasks simultaneously. In general, work overload and the extension of working hours is a reality in many occupations and sectors, and it is not possible that this can easily be changed [2].

According to the work demands-resource model by Bakker and Demerouti [18,19], job characteristics can be classified into two dimensions: demands and resources. This study focuses on demands, especially the way in which they have increased as a result of the acceleration of economic, social and technological changes worldwide. Franke [20] proposes that the phenomena associated with acceleration can be considered work overload. Therefore, it is presumed that the tools that facilitate this acceleration process to obtain agile solutions, such as the use of technologies, would also have implications for psychological distancing as part of the recovery from work-related stress. The following section details the location of the use of technologies as part of the theoretical proposal for this study.

### 1.3. Psychological Detachment from Work

Sonnentag and Bayer [15] propose the construct of psychological detachment from work and define it as the need to "mentally decompose yourself from work once the workplace is left behind" (p. 395). The only model to integrate this construct so far has been the stressor-detachment model by Sonnentag and Fritz [21], which considers detachment to be the central mechanism influencing the health and welfare of workers, depending on how it is managed.

In general, psychological detachment from work is identified as the central experience of recovery from work stress, according to studies in organizational psychology [15,21,22]. Recovery refers to the restoration process in which the level of attrition that has increased in response to a stressor (or any demand from work) returns to the previous level in an individual [5,23]; this can be seen as a process opposite to the wear process [24]. The literature recognizes the psychological experiences underlying the recovery process as important and of great influence on the levels of physical and psychological well-being of people facing high levels of stress in their work [25]. However, detachment is the central axis of the resource restoration process [21]. 

The present study addresses the relationship between the use of technology and psychological detachment from work as a result of work overload. Consistent with the mediation model undergoing empirical testing, the results indicate a positive relationship between the use of technology and perceived overload. Additionally, there is a negative relationship between work overload and levels of psychological detachment. The structure of the study continues with a second section in which the method used to obtain the results is presented, followed by a third section that expands on the aforementioned results. Finally, a discussion and conclusions are presented that seek to broaden the understanding of the stressor-detachment model.

**Figure 1:**
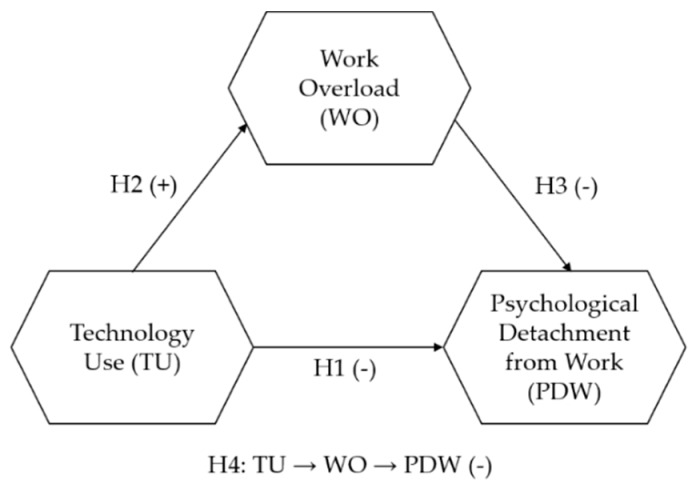
Research model and hypotheses. TU = technology use; WO = work overload; PDW = psychological detachment from work.

The stated objective allows us to ask the following research question: What is the effect that technology has on psychological detachment as a result of work overload? This leads to the establishment of the following research model (Figure 1) and hypotheses:

**Hypothesis 1** **(H1).** *There is a negative relationship between technology use (TU) and psychological detachment from work (PDW) Appendix A.*

**Hypothesis 2** **(H2).** *There is a positive relationship between technology use (TU) and work overload (WO).*

**Hypothesis 3** **(H3).** *There is a negative relationship between work overload (WO) and psychological detachment from work (PDW).*

**Hypothesis 4** **(H4).** *Work overload (WO) mediates the relationship between technology use (TU) and psychological detachment from work (PDW) (indirect effect).*

## 2. Materials and Methods

### 2.1. Participants

The database was obtained through a non-probability sampling of an intentional type, meaning the voluntary participation of our subjects. The optimal sample size was calculated using a prior statistical power analysis. The power of a statistical test is defined by its probability of rejecting a null hypothesis when in fact that hypothesis is false or has the potential for avoiding a Type II Error. The power analysis was executed using G*Power 3.1.9.4 software [26]. The calculation of the minimum sample size was performed based on the level of significance, the desired statistical power and the effect size expected. The level of significance was set at 0.05, the a priori statistical power was 0.80 (the minimum recommended in behavioral and health sciences), and the effect size [27]—in consideration of the smallest effect size of interest (SESOI)—was *f*^2^ = 0.02 [28]. The analysis revealed that a minimum sample size of 305 was necessary to obtain a power of 0.80 with two predictors (structural evaluation model).

The sample consisted of 313 professionals from the health sector with direct full-time employment contracts who responded to three instruments in order to investigate the research constructs. The workers belonged to a private company in the health services sector in Bogota, Colombia. Regarding this type of population, several investigations focused on the general well-being of workers in this type of organization established negative effects of different factors associated with work stress.

In relation to the characteristics of the sample, 239 were women (76%). Regarding the position held by the participants, 137 were technicians (44%), 85 were professionals (27%), 30 were area coordinators (10%) and 61 were heads or area managers (19%). Finally, 138 participants had employees of whom they were in charge (44%), while 224 professionals worked in administrative and management support areas (72%).

### 2.2. Instruments

#### 2.2.1. Measurement Scale of the Organization’s Demands for Detachment

The scale was developed by Sandoval [29] to measure the organization’s demands and resources for detachment. This scale has five subscales, although only the one regarding technological use was used in this study. This subscale has five items utilizing a five-point Likert scale ranging from strongly disagree (1) to strongly agree (5). The technological use subscale is validated based on the contents of the test as reviewed by four expert judges. Likewise, the five items have high homogeneity indices, which is indicative of a high dimensional consistency.

#### 2.2.2. Intensification of Job Demands Scale

This instrument was developed by Kubicek, Paškvan and Korunka [30] and measures the intensification of work demands as a source of work stress across five scales. For this study, the scale corresponding to work overload (WO) was used, which consists of five items utilizing a Likert scale of five response options (1 = strongly disagree to 5 = strongly agree). The original psychometric study used values of reliability (α > 0.80) and validity (internal structure, discriminant and convergent).

#### 2.2.3. Recovery Experience Questionnaire

This questionnaire was development by Sonnentag and Fritz [24] to assess how individuals unwind and recuperate from work during leisure time. It is composed of four dimensions (psychological detachment, relaxation, mastery and control). The psychological detachment scale was used for this study, which is composed of three items utilizing a five-point Likert scale (1 = I do not agree at all to 5 = I fully agree). The scale used has shown appropriate evidence of validity and reliability as reported in the study of the development and validation of the instrument.

### 2.3. Procedure

The current study is based on primary data collected from the measurement scales in a health facility. The surveys were distributed by email to health professionals, with prior authorization from the managers of the health facility. There were 1125 online questionnaires distributed, with a total response rate of 28%. When working with a computerized test, the tabulation of the answers was carried out automatically as the surveys were filled out.

### 2.4. Data Analysis

This study employs a partial least squares structural equation modeling (PLS-SEM) approach using the SmartPLS 3.2.8 software. PLS-SEM is a multivariate technique that combines two statistical procedures on the one hand, and linear regression on the other, using a method of factor reduction to simultaneously estimate a set of interrelated dependency relationships. Among the advantages of the PLS-SEM model is the consideration of multicollinearity based on independent and dependent factors, which makes it superior to multiple regression methods. Finally, the bootstrapping method was used to examine the significance of factor loadings, path coefficients and variances.

## 3. Results

### 3.1. Assessment of Measurement Model

Our analysis begins with an assessment of the measurement models. Results show that all measures of reflective constructs presented good reliability and validity indicators (Table 1). More specifically, most loadings exceeded the threshold value of 0.708 (except TU_1, TU_2, WO_3 and WO_5), and the average variances extracted (AVE) were higher than the critical value of 0.50 for work overload (WO) and psychological detachment from work (PDW). Furthermore, all the construct reliabilities (i.e., construct reliability measure and composite reliability) had values above 0.70 [31]. Finally, the discriminant validity assessment, based on the heterotrait–monotrait ratio of correlations (HTMT) measure [32], shows that all the HTMT values were significantly lower than 0.85, thus supporting the measures’ discriminant validity (Table 2).

### 3.2. Assessment of the Structural Model

#### 3.2.1. Collinearity

In line with the structural model assessment standard procedure [31], we first assess the structural model for collinearity issues by examining the variance inflation factor (VIF) values of all the predictor constructs in the model. As all the VIF values except for PDW_2 were below the more conservative threshold of 3.3, we conclude that collinearity was not at critical levels.

#### 3.2.2. Significance and Relevance of the Path Coefficients

The results of the bootstrapping procedure with 10,000 samples without sign changes reveals that most of the structural model relationships were significant. Specifically, it can be seen in Table 3 that TU had a significant and meaningful effect on PDW (−0.407, *p* < 0.001) and WO (0.355, *p* < 0.001), whereas the impact of WO on PDW was much less pronounced (−0.206, *p* < 0.001).

#### 3.2.3. In-Sample Model Fit

**Figure 2:**
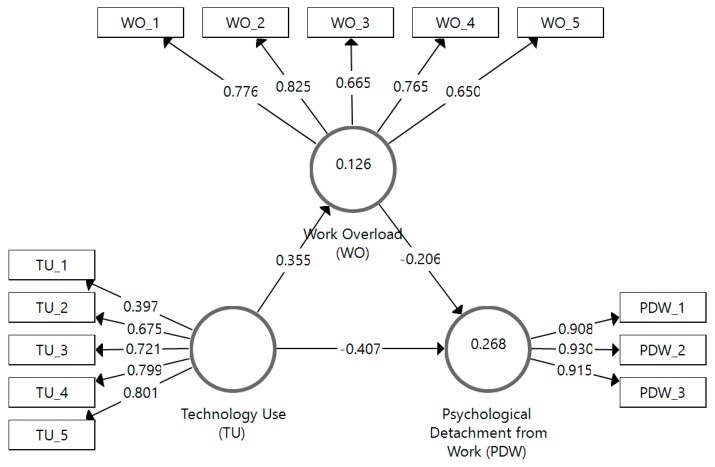
Assessment results of path coefficients.

To assess the model’s in-sample fit, we first consider the *R*^2^. It can be seen in Table 3 that endogenous constructs had *R*^2^ values below 0.30. While the model’s in-sample model fit was rather small according to absolute standards [31], we consider this acceptable for this study in light of it being a new model tested in a particular scenario (Figure 2).

#### 3.2.4. Out-of-Sample Predictive Power

We used PLSpredict with 10 folds and one repetition to mimic how the PLS model would eventually be used to predict new outcomes. Subsequently, we checked the *Q*^2^ to evaluate the predictive capacity of the research model. To interpret the *Q*^2^, positive values must be considered to indicate that any prediction error by the PLS model is smaller than a prediction error using the mean values. In order to achieve this evaluation, it is necessary to utilize the following prediction error statistics: mean square error (RMSE), mean absolute error (MAE) and percentage average absolute error (MAPE). The results in this study indicate that the model studied satisfies this criterion for the Psychological Detachment from Work (PDW) construct (Table 3).

## 4. Discussion

In support of the principles of the stressor-detachment model, this study explored and provided empirical evidence on the relationships among the use of technology at work and levels of psychological detachment from work. The study also addressed the mediating role work overload plays in the relationships among these variables. Additionally, our goals was to extend the literature regarding technology and its permanent use as a central axis of connectivity within the world of work, as well as its implications on the health and well-being of workers [21]. Likewise, we sought to identify, from a mediation approach, how technology that extends the demands of a workplace [14,33] is reflected in psychological detachment from work due to higher levels of work overload.

Our empirical findings support our hypothesis about a negative effect of the use of technology on psychological detachment through a significance coefficient. Equally, our hypothesis of a positive effect from technology use on work overload, and a negative effect of the reverse on psychological detachment, obtains empirical support through significance coefficient paths, but is less pronounced. Likewise, we found that there was a significant indirect effect of technology use on psychological detachment resulting from work overload, which provides evidence for acceptance of the fourth hypothesis of the present study.

These findings reinforce the proposed conceptual model and support the idea that being permanently connected through technological tools increases workloads, which in turn affects the possibility of reaching adequate levels of detachment [20]. In this way, the results obtained are consistent with previous findings that linked high workload levels with low levels of psychological detachment in different contexts [15,21]. The results also corroborated the relationship between the use of technologies such as smartphones and work–home interference, which was proposed by Derks and Bakker [17]. Our findings suggest that a greater implementation that work is intensifying more and more as a result of technologies in day-to-day work. Consequently, this also leads to a higher level of speed required, with more tasks to cover and less response time available. Although the phenomenon of technological acceleration seems to have no turning back [15], it is necessary to investigate this effect on the design and characteristics of jobs.

A theoretical contribution of our study is the development of the stressor-detachment model [21] and the job demands-resource model [18] by integrating the use of technology use in work environments as a management tool. We consider it extremely important for researchers to study how decisions to integrate technologies of communication and information impact the demands of a workplace and well-being of workers. More empirical research along this line will serve to enhance business and work external regulators policies that consider vulnerable-health indicators in the context of work overload [12,13].

At a practical level, our findings indicate that the use of technology as a management tool has an impact on people that should be taken into account by managers and organizational leaders [33]. One of the key points for managers to consider is the risks associated with work overload as a consequence of the implementation of technology. These risks are prevalent even when technology is implemented to facilitate job performance or connectivity among employees. In order to reach the agility and flexibility demanded by the current business climate, responsibilities are increased while management times and cycles are reduced. The decision to incorporate technologies that streamline organizational management seems to open doors to high connectivity business cultures; however, this likely lets people feel that a permanent connection to work is a natural thing, and technological tools may risk their physical and psychological well-being. In particular, studies on this subject are still very scarce in Latin America, and this study is one of the first contributions to the rising interest towards this subject in the region.

A very widespread present-day event in the business world is the decision to provide workers with mobile devices so that they can be in permanent contact with the affairs of the organization and give effective answers to opportunities and problems. The inevitable consequence of this decision is that regular workdays are extended, and people remain tied to the world of work beyond their base schedule unless corrective measures are taken. Thus, it is necessary to assess the whole picture of the impact that implementing a new technology may have, not only for the sake of productivity outcomes, but also for the maintenance of adequate physical and psychological states [6,7].

Therefore, we believe that managers should seek to strengthen clear boundaries to ensure that the segmentation of work and personal life after the workday has finished. Cultural policies and norms promoting less (or any) communication related to work issues after workdays will help employees to reach the right distances and adequate levels of recovery. The role of a manager does not only focus on the definition of standards, there is also a need for a manager to perform as a model for their employees in the way that technology is used as a resource rather than as a demand of the workplace [8]. Training middle managers to be responsible for modeling behaviors towards the use of technology and the balance between work and personal life may be a positive measure [1,3,9,20].

A limitation of this was the use of self-reporting instruments as measurement of constructs related to personality, which allows a greater possibility of presenting the social desires of those examined [34]. However, we sought to control this bias by informing participants about the anonymity of their responses, applying evaluations in a standardized manner and identifying strange response patterns as atypical or extreme values in the database. Future research could use other types of instruments such as observation lists or semi-structured interviews. 

## 5. Conclusions

The objective of study was to explore the relationship between the use of technology and psychological detachment from work as a result of work overload. The four hypotheses presented through the theoretical review of this study were contrasted and confirmed. PLS-SEM and PLSpredict methods were used in order to achieve the study objective.

According to results, technology has a negative effect on psychological detachment from work. On the other hand, the use of technology has a positive effect on work overload, which in turn has a negative effect on psychological detachment from work. Likewise, we found a significant indirect effect of work overload mediating the relationship between the use of technology and psychological detachment from work.

This study opens the social science debate on the theoretical–practical analysis of the implications of using technology in the health sector, where its inappropriate use would have negative consequences on professional staff such as work overload and failure to achieve a psychological detachment from work. It also guides the generation of new research lines that deepen the impact of the use of technology within institutions in other different sectors. Further research should focus on looking for what other variables mediate the negative impact of the use of technology on psychological detachment from work, to propose better-integrated intervention plans to enhance workers’ well-being.

## Figures and Tables

**Table 1:** Assessment of convergent validity and internal consistency reliability.

Construct/Indicators	Outer Loadings	Weights	VIF	rho_A	CR	AVE
Technology use (TU)				0.781	0.816	0.482
TU_1	0.397	0.068	1.210			
TU_2	0.675	0.277	1.435			
TU_3	0.721	0.292	1.431			
TU_4	0.799	0.317	1.727			
TU_5	0.801	0.403	1.617			
Work overload (WO)				0.807	0.857	0.547
WO_1	0.776	0.319	1.579			
WO_2	0.825	0.271	2.050			
WO_3	0.665	0.250	1.348			
WO_4	0.765	0.312	1.527			
WO_5	0.650	0.190	1.491			
Psychological detachment from work (PDW)				0.907	0.941	0.842
PDW_1	0.908	0.368	2.664			
PDW_2	0.930	0.375	3.307			
PDW_3	0.915	0.347	3.028			

Note: VIF = variance inflation factor; rho_A = construct reliability measure; CR = composite reliability; AVE = average variance extracted.

**Table 2:** Assessment of discriminant validity using the heterotrait–monotrait ratio (HTMT).

Construct	Technology Use (TU)	Work Overload (WO)	Psychological Detachment from Work (PDW)
Technology use (TU)			
Work overload (WO)	0.422 [0.300; 0.535]		
Psychological detachment from work (PDW)	0.528 [0.403; 0.631]	0.401 [0.289; 0.507]	

Note: Numbers in brackets represent the 95% bias-corrected and accelerated confidence intervals derived from bootstrapping with 10,000 samples.

**Table 3:** Structural model results and predictive performance summary.

Hypothesis	Path Coefficient	*t*-Statistic	*p*-value	95% BCCI	R^2^	Q^2^
H1 (TU → PDW)	−0.407	7.714	0.000	[−0.504; −0.296]	0.268	0.005
H2 (TU → WO)	0.355	7.351	0.000	[0.248; 0.438]	0.126	−0.273
H3 (WO → PDW)	−0.206	3.988	0.000	[−0.302; −0.096]		
H4 (TU → WO → PDW)	−0.073	3.621	0.000	[−0.114; −0.035]		

Note: 95% BCCI = 95% bias-corrected confidence intervals.

## Appendix A

Measurement items

1. Technology Use (TU)
  - There are different technological tools that assure others can contact me quickly and easily. (TU_1)
  - My responsibilities force me to continually use available technological tools (cell phone, email, chat, video conferencing). (TU_2)
  - I have downloaded work-related applications on my personal mobile devices. (TU_3)
  - It is expected that I will always be connected to work issues beyond my workday. (TU_4)
  - They contact me about work issues after my workday through my available mobile devices. (TU_5)
2. Work Overload (WO)
  - It is increasingly rare to have enough time for work tasks. (WO_1)
  - It is increasingly hard to take time for breaks. (WO_2)
  - The time between the more intense work phases has decreased. (WO_3)
  - One has to do two or three things at once (such as eating lunch, writing emails, and talking on the phone) more often. (WO_4)
  - A growing amount of work has to be completed by fewer and fewer employees. (WO_5)
3. Psychological Detachment from Work (PDW)
  - I forget about work. (PDW_1)
  - I don’t think about work at all. (PDW_2)
  - I distance myself from my work. (PDW_3)
